# Sufficient Social Support as a Possible Preventive Factor against Fighting and Bullying in School Children

**DOI:** 10.3390/ijerph15050870

**Published:** 2018-04-26

**Authors:** Kastytis Šmigelskas, Tomas Vaičiūnas, Justė Lukoševičiūtė, Marta Malinowska-Cieślik, Marina Melkumova, Eva Movsesyan, Apolinaras Zaborskis

**Affiliations:** 1 Health Research Institute, Faculty of Public Health, Medical Academy, Lithuanian University of Health Sciences, 47181 Kaunas, Lithuania; kastytis.smigelskas@lsmuni.lt (K.Š.); apolinaras.zaborskis@lsmuni.lt (A.Z.); 2 Department of Health Psychology, Faculty of Public Health, Medical Academy, Lithuanian University of Health Sciences, 47181 Kaunas, Lithuania; juste.lukoseviciute3@gmail.com; 3 Department of Environmental Health, Faculty of Health Sciences, Jagiellonian University Medical College, 20 Grzegorzecka Str., 31-351 Krakow, Poland; marta.malinowska-cieslik@uj.edu.pl; 4 Arabkir Medical Centre, Institute of Child and Adolescent Health, Yerevan 0014, Armenia; mmelkumova@mail.ru; 5 Arabkir Medical Centre, Institute of Child and Adolescent Health, 30 Mamikoniants Str., Yerevan 0014, Armenia; eva_mov@yahoo.com

**Keywords:** social support, bullying, fighting, adolescents

## Abstract

*Background:* This study aims to explore how sufficient social support can act as a possible preventive factor against fighting and bullying in school-aged children in 9 European countries. *Methods*: Data for this study were collected during the 2013/2014 Health Behaviour in School-aged Children (HBSC) survey. The sample consisted of 9 European countries, involving 43,667 school children in total, aged 11, 13 and 15 years. The analysed data focus on social context (relations with family, peers, and school) as well as risk behaviours such as smoking, drunkenness, fighting and bullying in adolescents. The relationships between social support and violent behaviour variables were estimated using multiple regression models and multivariate analyses. *Results*: Bullying, across 9 countries, was more prevalent than fighting, except for Armenia, Israel, and Poland. The prevalence among countries differed considerably, with fighting being most expressed in Armenia and bullying—in Latvia and Lithuania. The strongest risk factors for bullying and fighting were male gender (less expressed for bullying), smoking and alcohol consumption. In addition, for bullying the social support was similarly strong factor like above-mentioned factors, while for fighting—less significant, but still independent. All forms of social support were significantly relate with lower violent behaviour of school children, and family support was associated most strongly. Regardless the socioeconomic, historical, and cultural differences among selected countries, the enhancement and reinforcement of the social support from possible many different resources should be taken into consideration in prevention programs against school violence behaviours.

## 1. Introduction

Bullying and fighting at school are very common concerns of peer violence among adolescents in many international studies [1,2,3]. Bullying is defined as a “specific type of aggression in which the behavior is intended to harm or disturb, the behavior occurs repeatedly over time, and there is an imbalance of power, with a more powerful person or group attacking a less powerful one” [4]. The World Health Organization states that youth violence is as a result of an interaction between individual and social factors, especially relationships with family, peers, and teachers [5].

The 2006 UNESCO study on violence against children reported that 20–65% of schoolchildren are affected by verbal bullying and it is the most prevalent form of violence at school [6]. The existence of bullying at school has a negative impact on the school climate and violates the rights of children to learn in a safe environment without fear. Among adolescents, the most common risk factors for bullying perpetration are younger age, male gender, substance use and other health-threatening behaviours [7].

Studies show multiple predictors of bullying (not necessarily considered as causes), and individual characteristics and social contexts are crucial [8,9,10,11,12]. Family and home environment, school climate, and community factors significantly predict involvement for bullies, indicating the important role social context plays in the development and maintenance of bullying [13]. Specifically, the perceived emotional support from parents, peers, and teachers is associated with teenagers’ aggressive behaviours [7,14,15] and the primary source of social support for adolescents varies as a function of age—in early adolescence, parental support is important but in late adolescence, peer support becomes more salient [16,17]. Studies analyzing parents as a social influence show that greater parental warmth and support associates with less involvement in all forms of adolescent bullying [18,19]. Bullying perpetration and fighting are largely studied as a school-based phenomenon, so in this context the school climate and relationships with classmates and with teachers play significant roles in a child’s social support network, providing social support in its multiple forms [20].

Bullying is a “group process” at the peer-group level, and adolescents belonging to bullying groups increase their aggressive behaviours overtime through reciprocal rewarding and reinforcing of each other’s behaviours. Therefore, bullies who are dominant very often perceive themselves as popular among classmates and other peers [21]. At schools where bullying is a problem, children do not feel accepted, supported, respected, or fairly treated [2,22]. However, peer support is not the only relevant factor of support. Studies show that active teacher support is related to lower engagement in pupils’ health risk and antisocial behaviours such as bullying and fighting [23,24]. Teachers and school staff are in a unique and influential position to promote healthy relationships and to intervene in violent situations [25].

Even though it has been showed that the main predictors of bullying are delinquent behaviours as a risk factor and good social climate at school as a protective factor, the strong social bonds in the community are also supportive and can reduce the level of bullying at schools [26]. Students feeling more connected to their schools demonstrate reductions in violent behaviour over time [10]. Therefore, it can be seen that social factors are relevant in the onset and prevention of aggressive behaviours, though other individual behavioural factors (such as alcohol consumption or smoking) can also be among indicators associated with bullying or fighting. There is a need to study the role of perceived social support, besides the influence of individual factors, in planning and implementing youth violence prevention programs, also at the country level [3,7,13]. Therefore, our study was aimed to investigate how different factors play together in the occurrence of aggressive behaviours at school. For this, we analysed individual and social factors as well as main health risk behaviours in nine selected countries.

## 2. Methods

### 2.1. Data Collection

Data for this study were collected during the 2013/2014 Health Behaviour in School-aged Children (HBSC) survey, which is the collaborative project of World Health Organization Regional Office for Europe. The analysed data focus on social context (relationships with family, peers, and school teachers) as well as risk behaviours such as smoking, drunkenness, fighting and bullying in adolescents. Data were collected by school-based surveys using a standard international study protocol [27]. The samples at national levels were representative for the age groups of 11-, 13- and 15-year-olds attending school in each country and region. Cluster probability sampling (systematic or random) of school classes was carried out in each country and region. Sampling of schools was carried out where lists of classes were not available, followed by sampling of classes within schools. Each country data records were revised by the HBSC Data Management Centre at the Department of Health Promotion and Development, University of Bergen, Norway.

Appropriate ethical approval was granted in all countries and regions (approval code). Standardised information about the study was provided to parents and school children with the invitation to participate. Where possible, special adjustments were made to accommodate pupils who could not complete the questionnaire under standard conditions (through provision of, for instance, large-print versions or a reader).

### 2.2. Sample

The analysed sample consisted of 9 countries (Armenia, Estonia, Israel, Latvia, Lithuania, Moldova, Poland, Russia, and Ukraine), involving 43,667 school children in total. The samples within the countries ranged from 3679 to 6193 participants. Detailed characteristics of study sample by age, gender, and country are presented in Table 1.

### 2.3. Instruments

School support was assessed using the amended Teacher and Classmate Support Scale [28]. *Teachers support* was evaluated using 3 question items: “I feel that my teachers accept me as I am”, “I feel that my teachers care about me as a person”, and “I feel a lot of trust in my teachers”. Response options ranged from 1—“very strongly disagree” to 5—“strongly agree” for both teacher and classmate scales. *Classmates support* scale consisted of 3 items: “The students in my class(es) enjoy being together”, “Most of the students in my class(es) are kind and helpful”, and “Other students accept me as I am”. For analyses, the mean scale score for both teacher and classmate support scales was dichotomised: values from 1 to 2 pts were considered as “Low support”, and a score higher than 2 as “High support“. Confirmatory factor analysis from a number of the European countries [28,29] supported a two factor structure for these support scales and confirmed test-retest reliability and measurement invariance across countries.

Family and friends support was measured using the Multidimensional Scale of Perceived Social Support (MSPSS) [30]. The MSPSS has been well-validated and used in multiple studies and across different cultural contexts [31,32,33]. *Friends support* scale was evaluated using 4 items: “My friends really try to help me”, “I can count on my friends when things go wrong”, “I have friends with whom I can share my joys and sorrows”, and “I can talk about my problems with my friends”. Response options for both friend and family support ranged from 1—“very strongly disagree” to 7—“very strongly agree”. Dichotomisation of this scale was defined using the scale response descriptors, where mean scale scores ranging from 5 to 7 pts were considered as “High support”, and a score lower than 5 as “Low support”. *Family support* was evaluated by 4 items from the family subscale: “My family really tries to help me”, “I get the emotional help and support I need from my family”, “I can talk about my problems with my family”, and “My family is willing to help me make decisions”. Dichotomisation of family support scale was defined using the scale response descriptors, where mean scale scores ranging from 1 to 2 were considered as “High support”; a score lower than 1—categorised as “Low support”. Lithuania used different scale intervals (according to different response options), where the mean scale scores higher than 5 pts were considered as “High support”, and scores lower than 5 as “Low support“.

The internal consistency of all support scales across countries was acceptable, being 0.72 at lowest. The Cronbach alphas were consistently higher for family support (0.86–0.95) and friends’ support (0.80–0.93), indicating not only necessarily higher consistency, but higher number of items as well. Detailed data on internal consistency are presented in Table 2.

For overall social support analysis, the four scales were composed to one composite indicator—Overall Social Support Score. This score ranged from 0 to 4, indicating how many support types were high for every particular participant under study. Sufficient social support was considered as 3 or 4 points on Overall Social Support Score.

The Family Affluence Scale (FAS-III) [34] is an indicator of young people’s socio-economic status comprising six items on material assets in the family: “Does your family own a car, van or truck?”, “Do you have your own bedroom?”, “During the past 12 months, how many times did you travel away on holiday with your family?”, “How many computers does your family own?”,“How many baths/showers are there in your house”, and “Does your family have a dishwasher at home?” Scale scores were calculated by summing up the scores of all six items. A measure of relative FAS (proportions of low-medium-high in each country) of 20-60-20 percentiles was used in the current study, according to analysis applied in the HBCS international report from the 2013/2014 survey [35]. Armenia and Lithuania did not include two and one FAS items, respectively, lowering the theoretically maximum scale score; nevertheless, the FAS subgroups for those countries were also based on 20-60-20 percentiles of their distributions (Table 3).

*Fighting* was evaluated with the question: “During the past 12 months, how many times were you involved in a physical fight?” Responses of at least 3 times in the last 12 months have been classified as frequent physical fighting and a dichotomous variable was constructed in line with this [36].

To measure *bullying perpetration*, adolescents were asked “How often have you taken part in bullying another student(s) at school in the past couple of months?” These questions were preceded by a definition of bullying [37], which has been well-used and validated in empirical studies in multiple countries [38,39]. Reports of at least 2 or 3 times a month in the past couple of months have been considered as chronic bullying [40,41].

*Smoking* was evaluated through the question adapted from Monitoring the Future Study and the European School Survey Project on Alcohol and Other Drugs (ESPAD) [42]. Participants answered on a 4-point Likert scale from 1—“never” to 4—“every day”. A dichotomous variable of smoking was created by recoding (“rarely or never” or “at least once a week”). Self-report smoking measures have been found to have good reliability and validity [43].

*Drunkenness* was measured by the following question: “Have you ever had so much alcohol that you were really drunk?” Based on Swiss ESPAD data, the question on subjective drunkenness better measures risky single occasion drinking among adolescents rather than by asking about the frequency [44]. This item has been well used and found to have a good predictive and criterion validity [44]. As in previous papers using HBSC data, a dichotomous variable was created in order to identify adolescents involved in problematic alcohol use (indicating “never or once in the past month” or “twice or more”) and due to the skewed nature of the distribution [45].

The majority of the measures were dichotomised in order to have comparable odds ratios across different factors. In cases where different factors would have a different number of response categories, the differences will be more striking due to larger distances between extreme categories.

### 2.4. Statistical Analysis

The study data were analysed using the “IBM SPSS 20.0” statistical package. The descriptive analyses included means ± standard deviations (SD) as well as percentages. Inferential analysis was based on logistic regression. First, univariate regression was conducted to check the associations between analysed factors and outcomes (fighting or bullying). In multivariate regression, separate models were built to predict higher levels of proneness to fighting and bullying. The latter two were considered as binary outcomes. Putative risk factors included in multivariate models were perceived support from teachers, friends, family, and peers, sociodemographic indicators (gender, age group, and family affluence), and risk behaviours (smoking and drunkenness).

The strength of associations was expressed in odds ratios (OR). For inferential analysis, separate support scales were used to calculate an Overall Social Support Score (OSSS), reflecting on how many of social supports scales a particular adolescent had high social support. This was conducted due to strong associations between different types of support (odds ratios ranging at levels of 2 to 3 and more) that later in multivariate analyses may give underestimated associations due to strong interactions. Since we analysed four types of support, the OSSS score ranged from 0 (none of the supports is high) to 4 (all analysed social supports are high). For countrywise comparisons this was further dichotomised to an indicator of Sufficient Social Support, referring to the proportion of the population with at least 3 types of social support rated as “high”.

The multiple regression models were run separately for all countries together and for every country under analysis to check for possible (in)consistencies of risk effects. The statistical significance level was set at 5%.

## 3. Results

### 3.1. Descriptive Analysis

This study analysis included nine countries, mainly from Eastern Europe (Armenia, Estonia, Latvia, Lithuania, Moldova, Poland, Russia, and Ukraine) and included Israel as well. The bullying in the analysed countries was more prevalent than fighting, with the exception of Armenia, Israel, and Poland. The prevalence among countries differed several times, with fighting being most expressed in Armenia (19%) and bullying—in Latvia and Lithuania (23%). Current smoking was most prevalent in Russia (almost 9%), while drunkenness—in Lithuania (16%). The detailed information on the prevalence of abovementioned behaviours is presented in Table 4.

The social support in this study was analysed assessing four types of support—classmates, family, friends, and teachers support (Table 5). In most countries, it was high family support that was most expressed among different support types. It can also be noted that high friend support was usually more prevalent compared to classmate support, while the levels of the latter were similar to the teachers support.

For further analyses the social support was regarded as a total score composed of classmates, family, friends, and teachers support (as overall social support—OSSS). OSSS indicated the number of different support types that were rated as high. It was found that the highest prevalence of full social support (i.e., all four types of social support are high) was in Armenia (36%), Israel and Moldova (33% each).

For country wise comparisons additionally, we used a dichotomous social support measure—Sufficient Social Support, indicating how many participants had at least three of four analysed types of social support rated as high.

### 3.2. Overall Support as a Predictor of Fighting

Further, the analysis of potential risk and protective factors for fighting was conducted. For this, the univariate model was calculated first, with later adjustments for sociodemographic factors and risk behaviours. The findings suggest (Table 6) that the strongest risk factor for fighting was male gender: boys are 4.9 times more likely to have been involved in fighting than girls. A younger age was also associated with a higher risk of fighting. Among other independent risk factors for fighting, it was found that smoking and the experience of being drunk were both associated with a double likelihood of being involved in fighting.

Social support was also found as an independent factor associated with fighting (Table 6). In multivariate analysis, we used OSSS and it was found that the less social support, the higher the risk of fighting among adolescents: the respondents who had the lowest level of overall social support were 1.5 times more likely to have been involved into fighting compared to school children who had the highest perceived social support.

### 3.3. Overall Social Support as a Predictor of Bullying

The analysis of risk factors for bullying revealed (Table 7) that male gender and risk behaviours such as smoking or being drunk were significant predictors of bullying: these three factors were associated with an approximately double risk of bullying among school children. However, differently from fighting, gender had a less expressed effect.

On the other hand, the social support’s potential effect on bullying showed almost a linear pattern—the increase of one additional high support type was related with a quite similar increase of risk estimate (Table 7). In particular, a high risk of bullying was found for adolescents who reported the absence of high social support from all—family, friends, classmates, and teachers (OR = 3.1). This was the most vulnerable subgroup independent of gender, risk behaviours and other factors in the model.

### 3.4. Effects of Specific Types of Social Support on Aggressive Behaviours

In order to see specifically if different types of social support act similarly in predicting the likelihood of fighting and bullying, we analysed them in a multivariate model. The results show (Table 8) that in the case of fighting it was the high family support that acted most strongly (OR = 1.3), with the slightly lower relevance of teacher support (OR = 1.2). However, age and age-mates support (classmates and friends) do not seem to have a protective or risk effect against fighting. Regarding bullying, the high support of family was also dominant in predicting the risk of bullying, and more so than in case of fighting (OR = 1.5). The school-related support (by classmates and teachers) was also significant, though a slightly weaker predictor of bullying than family support.

### 3.5. Protective Effects of Social Support by Country

We established that there is an overall pattern that the social support seems to act as a protective factor against fighting and bullying almost in a linear manner (Table 6 and Table 7). Therefore, for further analyses on the countries comparison, we analysed the social support as a continuous variable with the score ranging from 0 (none of the four analysed social support scales indicating high support) to 4 (all four social support scales indicating high support). We found that social support as a potential protective factor acts specifically depending on the country.

The results showed (Table 9) that the strongest protective effect of overall social support against fighting was found in Israel and Poland (OR = 0.8) and differed significantly from the overall estimate (OR = 0.88; 95% CI 0.86–0.91). These two countries had the prevalence of fighting as one of the lowest among analysed countries. Exceptionally, the data showed that the country with the highest prevalence of fighting, i.e., Armenia, does not face a protective effect from social support (OR = 1.02), unlike other analysed countries.

The most expressed protective effect of social support against bullying was found in Estonia and Israel (OR = 0.7); however, the difference from the overall estimate was non-significant. Of note, these countries had higher than average prevalence of sufficient social support and lower prevalence of bullying. Almost the opposite was revealed to occur in countries with the highest prevalence of bullying—Latvia and Lithuania.

## 4. Discussion

The public health approach to the problem of violent behaviours among adolescents seeks to identify not only the risk but also protective factors in order to determine when in the lifespan they occur and how to develop the prevention programs [46,47]. It is a complex phenomenon that involves the social framework, relationships within the family, friends, and teachers [8,11,48].

There are complex interactions of individual and social determinants [12] predicting that the adolescent becomes violent. The underlying predictors of school violence and bullying include gender and social norms and wider contextual and structural factors [11,49]. For example, in some Eastern countries, boys are encouraged to be stronger, more aggressive and even fight to prove their masculinity; the social norms existing in many communities in those countries do not support males being too gentle and peaceful. It might be partially caused by difficult historical situation and many wars experienced in the past history and other factors.

Current survey data, in general, proved the idea published in the report of UNESCO in 2017 that physical violence in industrialised countries is less common in schools than bullying [50]. Although the individual characteristics and dynamics that underlie social value and power may vary across cultures and countries, the abuse of power to distress or control another person is consistently observed [51]. School violence and bullying are often invisible or ignored by teachers and parents. In some contexts, adults view physical punishment, fighting, and bullying as a normal part of discipline or growing up and are not aware of the negative impact they have on the education, health and well-being of children and adolescents [50].

Comprehensive assessments of children’s involvement in bullying and victimisation must take into account their relationships with parents since parents are the primary socialisation agents. Close relationships with adults, especially parental support and time spent with parents, are protective factors of bullying perpetration among adolescents [11,52], which is consistent with our study results. In our study, family support seems to act as the strongest potential protector against fighting and bullying. In the family context, some studies show that parental support is also negatively related to the perpetration of a physical fight and verbal, relational bullying [19]. In studies specifically focused on protective factors of bullying and victimisation [8,52], researchers have found that family support acts as a strong risk-based protective factor, especially when there is a strong attachment to parents in the family. Parents need support and encouragement to promote children’s social competencies and healthy relationships.

Though the family is usually considered as a primary factor for social support [49,52,53], in case of adolescent aggressive behaviours, it was found that teacher support may also play a significant role [52,53]. Levels of bullying are higher in schools where teachers use authoritarian and inflexible practices to cope with student misbehaviour [54]. Authoritarian practices of enforcing discipline distance the students from their teachers and decrease trust in them. However, both teachers and students report that teachers do not know how to effectively intervene, which prevents students from seeking help and contributes to teachers ignoring bullying [55]. Worsening relationships with teachers and disliking school increased the likelihood of violent behavior [18]. Therefore, it is essential to increase teachers’ awareness on how to act in order to prevent violent behaviours of adolescents. Moreover, this prevention should not be based only on direct prevention of bullying and fighting, but rather on improvement of social relationships between school children and teachers.

School bonding is related to both bullying perpetration and victimisation, with possible bi-directional influences [49]. Many studies show that peer support is positively related to substance use or risk behaviour [56,57] and is a protective factor for bullying behaviours [17,18]. As shown in this study data, the relationship between peers, teachers and bullying is complex, where received social support from each of these groups acts as a very strong and comprehensive protection factor. School staff and students require understanding about bullying and peers’ roles in promoting or preventing bullying. Having friends is a protective factor; therefore, children who are victimised benefit from supportive peer relationships. Promoting social skills and attitudes that are supportive of victimised youth is critical to creating a climate in which peers will intervene.

In line with previous studies [58] the present study also showed that risk behaviours such as smoking or being drunk were significant predictors of bullying. Risk behaviors include substance use, and aggression and violence have negative effects both on adolescents, their families and community [59].

In our study, the social support was found to be multidimensional—each form of social support was significantly related to lower levels of violent behaviour, but also the more different forms of social support were associated with lower involvement in aggressive behaviours. Dose-response relationships found in our study when talking about overall social support versus fighting and bullying indirectly may indicate ahigher likelihood of causality based on Hill’s causality criterion of biological gradient [60,61]. Our results show that overall social support as a potential protective factor acts specifically depending on the country, and it was pattern that countries, which have lowest rates of bullying and fighting, differed significantly in overall social support (much higher than in other countries).

The HBSC survey data analysis on the example of nine selected countries demonstrates implied but not always verifiable truth that what fits the overall region or dataset does not necessarily fit particular countries or subsamples. This strongly depends on the homogeneity of risk estimates. Therefore, the conclusions based on large-scale merged international data of different countries should be re-addressed scientifically or at least dealt with some caution when it comes to implication at national levels. Nevertheless, the heterogeneity of risk estimates across countries should not serve as a suggestion to abolish or raise doubt on international or merged analyses—rather, in cases where overall estimates on the burden of disease or risk factors are needed, the merged datasets that are based on unified study protocols should be of primary relevance and reliability.

When talking about the limitations of this study, it should be noted that self-reported aggressive behaviours may not always reflect the true prevalence of such behaviours. On the other hand, the data are based on a rich dataset from different countries. Therefore, it provides an opportunity to examine possible protective effects of social support in a consistent manner across different social environments peculiar for specific countries.

An integrated approach to school violence prevention programs in schools with teachers, peers and parents involvement is needed to provide counselling and skills to cope with and resolve violence among pupils and to reduce engagement in smoking and alcohol consumption. Policy regulation development as well as building a health and safety promoting environment should effectively prevent violent behaviours among adolescents.

More research is needed to look at similarities and differences in the development of fighting and bullying, as well as potential social protective factors across cultures in order to identify how cultural factors may be related to bullying involvement, with a view to learning effective strategies to promote children’s social skills and healthy relationships. Also, research is needed on protective psychosocial and behavioural factors as well as factors related to fighting and bullying in countries with various cultural and social norms with a view to learning effective strategies to promote children’s social skills and healthy relationships. This suggests that in some cases the analysis of separate countries may be reasonable, together with overall international comparisons. In addition, our study provides an insight into the multicausality of aggressive behaviours among school children, where the social environment and support are likely to play a role in preventing or triggering these behaviours independently from such well-established risk behaviours like alcohol drinking or smoking.

## 5. Conclusions

With some exceptions, the majority of the study findings confirm the findings of other researchers. Bullying, across 9 countries, was more prevalent than fighting, with the exception of Armenia, Israel, and Poland. The prevalence among countries differed by several times, with fighting being most expressed in Armenia and bullying—in Latvia and Lithuania. The strongest risk factor for bullying and fighting was related with male gender (less expressed for bullying), smoking and alcohol consumption. High support by friends and teachers seems to be quite a strong protective factor for school childrens violent bahaviours. Thus, social support was found to be multidimensional and to act as a possible protective factor in an almost linear manner—the increase of one additional high support type (classmates, family, friends and teachers) was significantly related to lower levels of violent behaviour. Also, a number of various forms of social support was associated with lower involvement in aggressive behaviours. Regardless the socioeconomic, historical, and cultural differences among selected countries, the enhancement and reinforcement of the social support from possible many different resources should be taken into consideration in prevention programs against school violence behaviours.

## Figures and Tables

**Table 1:** Study sample by age and gender in analysed countries.

Country	*n*	Age Group			Gender	
		11 Years	13 Years	15 Years	Boys	Girls
Armenia	3679	40.0%	31.6%	28.4%	47.8%	52.2%
Estonia	4057	33.4%	35.3%	31.3%	50.3%	49.7%
Israel	6193	39.8%	30.1%	30.1%	48.7%	51.3%
Latvia	5557	33.5%	35.3%	31.2%	47.7%	52.3%
Lithuania	5730	35.2%	35.2%	29.6%	50.8%	49.2%
Moldova	4648	33.2%	33.3%	33.5%	50.5%	49.5%
Poland	4545	33.4%	33.8%	32.9%	49.8%	50.2%
Russia	4716	30.2%	38.2%	31.6%	43.8%	56.2%
Ukraine	4552	32.4%	30.4%	37.2%	47.4%	52.6%

**Table 2:** Internal consistency of support scales by country (Cronbach alphas).

Country	Classmates	Family	Friends	Teachers
Armenia	0.794	0.875	0.795	0.763
Estonia	0.790	0.945	0.950	0.820
Israel	0.755	0.921	0.890	0.890
Latvia	0.772	0.953	0.918	0.774
Lithuania	0.818	0.861	0.919	0.844
Moldova	0.761	0.914	0.882	0.769
Poland	0.742	0.939	0.920	0.847
Russia	0.777	0.925	0.923	0.830
Ukraine	0.804	0.880	0.932	0.720

**Table 3:** Family affluence scores across analyzed countries.

Country	Family Affluence Scale Scores		Family Affluence, pts		
	Mean ± SD	Mean as % of Maximum Score	Low	Medium	High
Armenia	3.7 ± 2.01	41.1	0–1	2–5	6–9
Estonia	7.5 ± 2.36	57.8	0–5	6–9	10–13
Israel	7.7 ± 2.49	59.1	0–5	6–9	10–13
Lithuania	6.2 ± 2.26	62.4	0–4	6–8	9–10
Latvia	6.5 ± 2.52	50.1	0–4	5–8	9–13
Moldova	5.2 ± 2.75	40.2	0–2	3–7	8–13
Poland	6.9 ± 2.47	52.9	0–4	5–9	10–13
Russia	6.2 ± 2.44	48.0	0–4	5–8	9–13
Ukraine	5.3 ± 2.32	41.0	0–3	4–7	8–13

**Table 4:** Prevalence of violent and health risk behaviours among school children by country.

Country	Fighting 3 or More Times in the Last 12 Months, %	Bullying 2–3 Times and More in the Past Couple of Months, %	Current Smoking at Least Once a Week, %	Drunkenness at Least Twice in Life, %
Armenia	19.1	4.8	1.8	7.4
Estonia	7.8	10.3	5.4	12.2
Israel	10.1	8.2	6.7	4.1
Lithuania	9.4	22.9	7.5	15.9
Latvia	10.9	23.3	5.8	11.9
Moldova	11.6	13.9	3.6	9.5
Poland	11.3	9.6	8.0	11.7
Russia	11.8	17.2	8.6	6.7
Ukraine	12.3	12.8	5.7	8.8

**Table 5:** Prevalence of high social support by country and support type, %.

Country	Type of Social Support				Overall Social Support Score					Sufficient Social Support
	Classmates	Family	Friends	Teachers	0	1	2	3	4	%
Armenia	78.3	84.0	77.8	70.3	10.4	9.7	21.9	22.1	35.9	58.0
Estonia	54.4	77.1	63.8	46.6	6.5	17.9	26.6	27.4	21.7	49.0
Israel	66.0	84.2	68.7	63.1	5.9	13.2	19.9	27.7	33.3	61.0
Lithuania	47.8	63.6	69.6	50.9	10.2	19.9	24.2	24.0	21.7	45.7
Latvia	48.9	67.2	54.9	53.7	10.1	19.3	28.5	23.5	18.5	42.0
Moldova	67.2	82.1	67.0	59.3	3.3	12.1	23.5	28.0	33.1	61.1
Poland	53.8	74.7	65.0	46.4	8.4	17.5	26.2	25.8	22.1	47.9
Russia	45.4	68.0	57.5	45.1	14.8	20.6	28.8	19.8	16.1	35.8
Ukraine	49.7	76.0	63.4	43.8	7.7	20.3	27.5	24.9	19.6	44.4

**Table 6:** Fighting and associated factors in all countries: logistic regression.

Characteristic	Prevalence, %	Model 1 *		Model 2 **		Model 3 ***	
		Odds Ratio	CI 95%	Odds Ratio	CI 95%	Odds ratio	CI 95%
Gender							
Girls	4.2	1.00		1.00		1.00	
Boys	19.0	5.32	4.94–5.73	5.18	4.80–5.59	4.93	4.56–5.33
Age							
15 years	9.6	1.00		1.00		1.00	
13 years	11.2	1.18	1.09–1.28	1.18	1.09–1.28	1.52	1.39–1.66
11 years	13.2	1.44	1.33–1.55	1.52	1.40–1.65	2.13	1.95–2.33
Family affluence							
Low	10.3	1.00		1.00		1.00	
Medium	10.7	1.04	0.96–1.13	1.01	0.93–1.09	1.04	0.96–1.14
High	13.4	1.35	1.23–1.48	1.26	1.14–1.39	1.29	1.17–1.43
Current smoking							
Rarely or never	10.5	1.00				1.00	
At least once a week	24.2	2.72	2.47–3.00			2.12	1.88–2.38
Drunkenness in lifetime							
Never or once	10.1	1.00				1.00	
At least twice	21.5	2.43	2.24–2.64			2.22	2.00–2.45
Classmates support							
High	10.9	1.00					
Average or low	11.5	1.06	1.00–1.13				
Family support							
High	10.3	1.00					
Average or low	13.7	1.39	1.30–1.48				
Friends support							
High	10.3	1.00					
Average or low	12.7	1.26	1.18–1.34				
Teachers support							
High	10.1	1.00					
Average or low	12.3	1.25	1.18–1.33				
Overall support							
4 high support types	9.1	1.00		1.00		1.00	
3 high support types	10.2	1.13	1.03–1.24	1.17	1.06–1.28	1.12	1.01–1.23
2 high support types	12.2	1.39	1.27–1.52	1.41	1.28–1.55	1.32	1.20–1.45
1 high support types	13.8	1.61	1.46–1.77	1.66	1.50–1.84	1.54	1.39–1.70
0 high support types	14.7	1.73	1.54–1.95	1.70	1.49–1.93	1.50	1.31–1.71

* Model 1—univariate regression. ** Model 2—multivariate regression, adjusted for gender, age, and family affluence. *** Model 3—multivariate regression, adjusted for gender, age, family affluence, smoking, and drunkenness.

**Table 7:** Bullying and associated factors in all countries: logistic regression.

Characteristic	Prevalence, %	Model 1 *		Model 2 **		Model 3 ***	
		Odds Ratio	CI 95%	Odds Ratio	CI 95%	Odds Ratio	CI 95%
Gender							
Girls	9.8	1.00		1.00		1.00	
Boys	19.2	2.17	2.05–2.30	2.18	2.06–2.31	2.05	1.94–2.18
Age							
15 years	15.4	1.00		1.00		1.00	
13 years	15.5	1.01	0.94–1.08	1.03	0.96–1.10	1.30	1.21–1.40
11 years	12.2	0.76	0.71–0.82	0.84	0.78–0.90	1.14	1.06–1.24
Family affluence							
Low	15.9	1.00		1.00		1.00	
Medium	13.6	0.83	0.77–0.89	0.86	0.80–0.92	0.87	0.81–0.94
High	14.7	0.91	0.83–0.99	0.94	0.86–1.02	0.94	0.86–1.02
Current smoking							
Rarely or never	13.2	1.00				1.00	
At least once a week	31.5	3.02	2.76–3.31			1.78	1.60–1.97
Drunkenness in lifetime							
Never or once	12.5	1.00				1.00	
At least twice	30.7	3.11	2.89–3.35			2.38	2.18–2.59
Classmates support							
High	11.7	1.00					
Average or low	17.6	1.61	1.53–1.71				
Family support							
High	12.1	1.00					
Average or low	20.9	1.93	1.82–2.04				
Friends support							
High	12.5	1.00					
Average or low	17.7	1.51	1.43–1.60				
Teachers support							
High	11.3	1.00					
Average or low	17.7	1.70	1.61–1.80				
Overall support							
4 high support types	8.1	1.00		1.00		1.00	
3 high support types	12.4	1.60	1.46–1.76	1.58	1.44–1.74	1.52	1.39–1.67
2 high support types	16.1	2.16	1.98–2.36	2.09	1.91–2.29	1.97	1.80–2.15
1 high support types	19.2	2.68	2.44–2.94	2.56	2.33–2.81	2.38	2.16–2.62
0 high support types	24.0	3.56	3.20–3.96	3.41	3.05–3.82	3.07	2.74–3.44

* Model 1—univariate regression. ** Model 2—multivariate regression, adjusted for gender, age, and family affluence. *** Model 3—multivariate regression, adjusted for gender, age, family affluence, smoking, and drunkenness.

**Table 8:** Different types of social support as protective factors against fighting and bullying.

Type of Support	Fighting			Bullying		
	Prevalence, %	Odds Ratio *	95% CI	Prevalence, %	Odds Ratio *	95% CI
Classmates						
High	10.9	1.00		11.7	1.00	
Average or low	11.5	1.05	0.98–1.13	17.6	1.37	1.28–1.46
Family support						
High	10.3	1.00		12.1	1.00	
Average or low	13.7	1.31	1.21–1.42	20.9	1.52	1.42–1.62
Friends support						
High	10.3	1.00		12.5	1.00	
Average or low	12.7	0.96	0.89–1.03	17.7	1.12	1.05–1.19
Teachers support						
High	10.1	1.00		11.3	1.00	
Average or low	12.3	1.24	1.15–1.33	17.7	1.32	1.24–1.41

* Multivariate logistic regression, adjusted for gender, age, family affluence, smoking, drunkenness, and other types of support.

**Table 9:** Overall social support as protective factor against fighting and bullying.

Country	Fighting		Bullying	
	Odds Ratio *	95% CI	Odds Ratio *	95% CI
Armenia	1.02	0.94–1.10	0.77	0.68–0.87
Estonia	0.80	0.72–0.88	0.69	0.63–0.76
Israel	0.78	0.72–0.85	0.70	0.64–0.77
Lithuania	0.91	0.84–0.98	0.82	0.78–0.87
Latvia	0.85	0.79–0.91	0.88	0.84–0.93
Moldova	0.84	0.77–0.91	0.77	0.71–0.83
Poland	0.78	0.72–0.85	0.77	0.71–0.85
Russia	0.92	0.85–1.00	0.78	0.72–0.83
Ukraine	0.89	0.82–0.96	0.77	0.71–0.84
All countries	0.88	0.86–0.91	0.76	0.75–0.78

* adjusted for age, gender, family affluence, smoking, and drunkenness.

## References

[1] Elgar F.J., McKinnon B., Walsh S.D., Freeman J., Donnelly P.D., De Matos M.G., Gariepy G., Aleman-Diaz A.Y., Pickett W., Molcho M. (2015). Structural determinants of youth bullying and fighting in 79 countries. J. Adolesc. Health.

[2] Juvonen J., Graham S. (2014). Bullying in Schools: The Power of Bullies and the Plight of Victims. Annu. Rev. Psychol..

[3] World Health Organization (2016). Global Status Report on Violence Prevention 2014.

[4] Nansel T.R., Overpeck M., Pilla R.S., Ruan W.J., Simons-Morton B., Scheidt P. (2001). Bullying Behaviors Among US Youth: Prevalence and Association with Psychosocial Adjustment. J. Am. Med. Assoc..

[5] World Health Organization (2015). Preventing Youth Violence: An Overview of the Evidence.

[6] United Nations (2006). Violence Against Children.

[7] Shetgiri R. (2013). Bullying and victimization among children. Adv. Pediatr..

[8] Baldry A.C., Farrington D.P. (2005). Protective factors as moderators of risk factors in adolescence bullying. Soc. Psychol. Educ..

[9] Espelage D.L., Bosworth K., Simon T.R. (2000). Examining the social context of bullying behaviors in early adolescence. J. Couns. Dev..

[10] Fraser M.W. (1996). Aggressive Behavior in Childhood and Early Adolescence: An Ecological-Developmental Perspective on Youth Violence. Soc. Work.

[11] Hemphill S.A., Smith R., Toumbourou J.W., Herrenkohl T.I., Catalano R.F., McMorris B.J., Romanuik H. (2009). Modifiable determinants of youth violence in Australia and the United States: A longitudinal study. Aust. N. Z. J. Criminol..

[12] Hong J.S., Espelage D.L. (2012). A review of research on bullying and peer victimization in school: An ecological system analysis. Aggress. Violent Behav..

[13] Cook C.R., Williams K.R., Guerra N.G., Kim T.E., Sadek S. (2010). Predictors of bullying and victimization in childhood and adolescence: A meta-analytic investigation. School Psychol. Q..

[14] Espelege D.L., Swearer S.M. (2003). Research on school bullying and victimization: What have we learned and where do we go from here?. School Psychol. Rev..

[15] Wilson D. (2004). The interface of school climate and school connectedness and relationships with aggression and victimization. J. School Health.

[16] Furman W., Buhrmester D. (1992). Age and Sex Differences in Perceptions of Networks of Personal Relationships. Child Dev..

[17] Holt M.K., Espelage D.L. (2007). Perceived social support among bullies, victims, and bully-victims. J. Youth Adolesc..

[18] Mann M.J., Kristjansson A.L., Sigfusdottir I.D., Smith M.L. (2015). The Role of Community, Family, Peer, and School Factors in Group Bullying: Implications for School-Based Intervention. J. School Health.

[19] Wang J., Iannotti R.J., Nansel T.R. (2009). School Bullying Among Adolescents in the United States: Physical, Verbal, Relational, and Cyber. J. Adolesc. Health.

[20] Flaspohler P.D., Elfstrom J.L., Vanderzee K.L., Sink H.E., Birchmeier Z. (2009). Stand by me: The effects of peer and teacher support in mitigating the impact of bullying on quality of life. Psychol. School.

[21] Salmivalli C. (2010). Bullying and the peer group: A review. Aggress. Violent Behav..

[22] Payne A.A., Gottfredson D.C. (2004). Schools and Bullying. Bullying.

[23] Brooks F.M., Magnusson J., Spencer N., Morgan A. (2012). Adolescent multiple risk behaviour: An asset approach to the role of family, school and community. J. Public Health.

[24] Tomé G., Gaspar M., Matos D., Camacho I., Simões C. (2014). Friendships Quality and Classmates Support: How to Influence the Well-Being of Adolescents. High Educ. Soc. Sci..

[25] Pepler D.J., Craig W.M., Connolly J.A., Yuile A., McMaster L., Jiang D. (2006). A developmental perspective on bullying. Aggress. Behav..

[26] Stalmach M., Tabak I., Radiukiewicz K. (2014). Selected family socio-economic factors as predictors of peer violence among school children in Poland. Dev. Period Med..

[27] Currie C., Inchley J., Molcho M., Lenzi M., Veselska Z., Wild F. (2014). Health Behaviour in School-Aged Children Protocol: Background, Methodology and Mandatory Items for the 2013/2014 Survey.

[28] Torsheim T., Wold B., Samndal O. (2000). The teacher and classmate support scale. Factor structure, Test-retest Reliability and Validility in Samples of 13- and 15-Year old Adolescents. School Psychol. Int..

[29] Freeman J.G., Samdal O., Klinger D.A., Dur W., Griebler R., Currie D., Rasmussen M. (2009). The relationship of schools to emotional health and bullying. Int. J. Public Health.

[30] Zimet G.D., Dahlem N.W., Zimet S.G., Farley G.K. (1988). The multidimensional scale of perceived social support. J. Pers. Assess..

[31] Canty-Mitchell J., Zimet G.D. (2000). Psychometric properties of the multidimensional scale of perceived social support in urban adolescents. Am. J. Community Psychol..

[32] Edwards L.M. (2004). Measuring Perceived Social Support in Mexican American Youth: Psychometric Properties of the Multidimensional Scale of Perceived Social Support. Hisp. J. Behav. Sci..

[33] Ng C.G., Amer Siddiq A.N., Aida S.A., Zainal N.Z., Koh O.H. (2010). Validation of the Malay version of the Multidimensional Scale of Perceived Social Support (MSPSS-M) among a group of medical students in Faculty of Medicine, University Malaya. Asian J. Psychiatry.

[34] Torsheim T., Cavallo F., Levin K.A., Schnohr C., Mazur J., Niclasen B., Currie C. (2016). FAS Development Study Group. Psychometric Validation of the Revised Family Affluence Scale: A Latent Variable Approach. Child Indic. Res..

[35] Inchley J., Currie D., Young T., Samdal O., Torsheim T., Augustson L. (2016). Growing up Unequal: Gender and Socioeconomic Differences in Young People’s Health and Well-Being. Health Policy for Children and Adolescents.

[36] Pickett W., Molcho M., Elgar F.J., Brooks F., de Looze M., Rathmann K., Ter Bogt T.F., Gabhainn S.N., Sigmundová D., de Matos M.G. (2013). Trends and Socioeconomic Correlates of Adolescent Physical Fighting in 30 Countries. Pediatrics.

[37] Olweus D. (1996). Bullying at school: Knowledge base and an effective intervention program. Annals of the New York Academy of Sciences.

[38] Due P., Holstein B.E., Lynch J., Diderichsen F., Gabhain S.N., Scheidt P., Currie C. (2005). Bullying and symptoms among school-aged children: International comparative cross sectional study in 28 countries. Eur. J. Public Health.

[39] Elgar F.J., Craig W., Boyce W., Morgan A., Vella-Zarb R. (2009). Income Inequality and School Bullying: Multilevel Study of Adolescents in 37 Countries. J. Adolesc. Health.

[40] Dube S.R., Fairweather D., Pearson W.S., Felitti V.J., Anda R.F., Croft J.B. (2009). Cumulative childhood stress and autoimmune diseases in adults. Psychosom. Med..

[41] Harel-Fisch Y., Walsh S.D., Fogel-Grinvald H., Amitai G., Pickett W., Molcho M., Due P., de Matos M.G., Craig W. (2011). Negative school perceptions and involvement in school bullying: A universal relationship across 40 countries. J. Adolesc..

[42] Johnston L.D., Malley P.M.O., Miech R.A., Bachman J.G., Schulenberg J.E. (2015). Monitoring the Future: Key Findings on Adolescent Drug Use.

[43] Rosenbaum J.E. (2009). Truth or consequences: The intertemporal consistency of adolescent self-report on the youth risk behavior survey. Am. J. Epidemiol..

[44] Gossrau-Breen D., Kuntsche E., Gmel G. (2010). My older sibling was drunk—Younger siblings’ drunkenness in relation to parental monitoring and the parent-adolescent relationship. J. Adolesc..

[45] Currie C., Roberts C., Morgan A., Smith R., Settertobulte W., Samdal O. (2002). Young people’s health in context Health behavior in School-aged Children (HBSC) study: International report from the 2001/2002 survey. Health Policy Child Adolesc..

[46] Bradshaw C.P. (2015). Translating research to practice in bullying prevention. Am. Psychol..

[47] Chalamandaris A.G., Piette D. (2015). School-based anti-bullying interventions: Systematic review of the methodology to assess their effectiveness. Aggress. Violent Behav..

[48] Wilson J.J., Administrator A., Hawkins J.D., Herrenkohl T.I., Farrington D.P., Brewer D., Catalano R.F., Harachi T.W., Cothern L. (2000). Predictors of Youth Violence. Aggress. Behav..

[49] Spriggs A.L., Iannotti R.J., Nansel T.R., Haynie D.L. (2007). Adolescent Bullying Involvement and Perceived Family, Peer and School Relations: Commonalities and Differences Across Race/Ethnicity. J. Adolesc. Health.

[50] United Nations Educational Scientific and Cultural Organization (2017). School Violence and Bullying: Global Status Report.

[51] Canada’s Authority on Research and Resources for Bullying Prevention. https://www.prevnet.ca/research/fact-sheets.

[52] Hemphill S.A., Tollit M., Herrenkohl T.I. (2014). Protective Factors Against the Impact of School Bullying Perpetration and Victimization on Young Adult Externalizing and Internalizing Problems. J. School Violence.

[53] Eşkisu M. (2014). The Relationship between Bullying, Family Functions, Perceived Social Support among High School Students. Procedia.

[54] Yoneyama S., Naito A. (2003). Problems with the paradigm: The school as a factor in understanding bullying (with special reference to Japan). Br. J. Sociol. Educ..

[55] Bauman S., Del Rio A. (2006). Preservice teachers’ responses to bullying scenarios: Comparing physical, verbal, and relational bullying. J. Educ. Psychol..

[56] Lifrak P.D., McKay J.R., Rostain A., Alterman A.I., O’Brien C.P. (1997). Relationship of perceived competencies, perceived social support, and gender to substance use in young adolescents. J. Am. Acad. Child Adolesc. Psychiatry.

[57] Piko B. (2000). Perceived Social Support from Parents and Peers: Which Is the Stronger Predictor of Adolescent Substance Use?. Subst. Use Misuse.

[58] Swahn M.H., Donovan J.E. (2004). Correlates and predictors of violent behavior among adolescent drinkers. J. Adolesc. Health.

[59] Simões C., Matos M.G. (2011). Offending, victimization, and double involvement: Differences and similarities between the three profiles. J. Cogn. Psychother..

[60] Crislip M. Causation and Hill’s Criteria 2010. https://sciencebasedmedicine.org/causation-and-hills-criteria/.

[61] Fedak K.M., Bernal A., Capshaw Z.A., Gross S. (2015). Applying the Bradford Hill criteria in the 21st century: How data integration has changed causal inference in molecular epidemiology. Emerg. Themes Epidemiol..

